# Domestic

**Published:** 1841-11-13

**Authors:** 


### DOMESTIC.

*Hyper-operating.*—We quote, under this head, the following from our cotemporary of the New York Medical Gazette, with whom we shall ever be happy to labour hand in hand in the combat against the prevailing vice of modern surgeons—*brilliant cutting and slashing!* There are hundreds who could remove, *with a grace*, a scrofulous enlargement of a parotid gland, and cut a carotid artery in doing so, but there are few who could share with the departed Sanson, one of the most sincere of Parisian chirurgical students, the well-deserved compliment contained in this article.

M. Larrey (son of the Baron) has recently extirpated the parotid gland, in a girl of nineteen, of scrofulous constitution, in whom the gland, after having been much enlarged, and then reduced to one-half by free cupping, became hard and smooth—presenting at one point fluctuation. M. Larrey concluded that this was cancer, or *would become cancer*, and determined, contrary to the advice of Biett, to remove it, *yielding to the entreaties of the patient and her friends.* He did remove it (cutting into the external carotid by the way)—the artery was secured, and the patient recovered. The tumour was presented to the Academy of Sciences, who satisfied themselves that it was really the parotid gland.

*Remarks.*—We put this operation on record as a piece of surgical history, but take leave to accompany it by the expression of our opinion, that a more unjustifiable operation could scarce be imagined. To extirpate the parotid under such circumstances, (a scrofulous patient and the gland beginning to suppurate,) was an operation, the idea of which was just worthy of the man who could blunder as M. L. did in cutting the carotid. The patient had a lucky escape from such hands.

To restore the good humour of those of our readers whom this piece of Barber Surgery may have disgusted, we translate a few lines from the eulogium lately delivered by Cruveilhier over Sanson—here we have the man of science speaking of and doing justice to another man of science.

"Than Sanson, no one was more profoundly impressed with this great truth, that *the perfection of surgery was the knowing how to avoid operations.* That an operation, even the most simple, is an extreme measure, which always endangers, more or less, the life of the patient, and which consequently, should only be practiced in cases of the most absolute necessity. His *surgical honesty* revolted against all those operations undertaken through complaisance, and all the so-called *bold operations*, which are not required and justified by clear indications."

Honour to the *surgical honesty* of Sanson—would that there was more of it in the world!

*Ed. N. Y. Med. Gaz.*

### HEALTH OF THE CITY.

INTERMENTS *in the City and Liberties of Philadelphia, from the 30th of October to the 6th of November.*

| Diseases. | Adults. | Children. | Diseases. | Adults. | Children. |
|---|---|---|---|---|---|
| Asphyxia, | 0 | 1 | *Brought forward,* | 35 | 22 |
| Apoplexy, | 1 | 0 | Intemperance, | 2 | 0 |
| Burns, | 1 | 0 | Marasmus, | 0 | 1 |
| Cancer, | 1 | 0 | Mania a Potu, | 1 | 0 |
| —— Womb, | 1 | 0 | Old age, | 4 | 0 |
| —— Stomach, | 1 | 0 | Palsy, | 1 | 0 |
| Croup, | 0 | 1 | Small pox, | 3 | 0 |
| Congestion of | | | Still-born, | 0 | 7 |
| brain, | 1 | 0 | Spitting of blood, | 1 | 0 |
| Consumption of | | | Tumours, | 1 | 0 |
| the lungs, | 12 | 3 | Unknown, | 2 | 1 |
| Convulsions, | 1 | 2 | | — | — |
| Dropsy, | 2 | 1 | Total, 81— | 50 | 31 |
| —— head, | 0 | 4 | | | |
| —— Breast, | 0 | 1 | | | |
| Disease of the Brain, | 1 | 0 | | | |
| Dysentery, | 3 | 0 | | | |
| Debility | 0 | 3 | | | |
| Fever, | 1 | 0 | | | |
| —— Congestive, | 2 | 0 | | | |
| —— Bilious, | 1 | 0 | | | |
| —— Typhus, | 2 | 0 | | | |
| —— Typhoid, | 1 | 0 | | | |
| —— Hectic, | 0 | 1 | | | |
| Hæmorrhage from Intestines, | 1 | 0 | | | |
| Inflammation of the Brain, | 0 | 1 | | | |
| —— Bronchi, | 0 | 1 | | | |
| —— Lungs, | 2 | 1 | | | |
| —— Bowels, | 0 | 2 | | | |
| | — | — | | | |
| *Carried forward,* | 35 | 22 | | | |

| Of the above, there were | |
|---|---|
| under 1 year, | 16 |
| From 1 to 2 | 2 |
| 2 to 5 | 8 |
| 5 to 10 | 2 |
| 10 to 15 | 1 |
| 15 to 20 | 2 |
| 20 to 30 | 14 |
| 30 to 40 | 12 |
| 40 to 50 | 6 |
| 50 to 60 | 5 |
| 60 to 70 | 6 |
| 70 to 80 | 4 |
| 80 to 90 | 3 |
| 90 to 100 | 0 |
| Total, | 81 |

Of the above there were 8 from the almshouse, 13 people of colour, and 1 from the country, which are included in the total amount.

